# Two Siblings with Isolated GH Deficiency Due to Loss−of−Function Mutation in the GHRHR Gene: Successful Treatment with Growth Hormone Despite Late Admission and Severe Growth Retardation

**DOI:** 10.4274/jcrpe.v2i4.164

**Published:** 2010-11-06

**Authors:** Zeynep Şıklar, Merih Berberoğlu, Maria Legendre, Serge Amselem, Olcay Evliyaoğlu, Bülent Hacıhamdioğlu, Şenay Savaş Erdeve, Gönül Öçal

**Affiliations:** 1 Ankara University, School of Medicine, Department of Pediatric Endocrinology, Ankara, Turkey; 2 Service de Genetique Medicale, Hopital Armand−Trousseau, Paris F−75012 France; Inserm U933, Hopital Armand−Trousseau, Paris F−75012 France; +90 505 342 21 69zeynepsklr@gmail.comAnkara University School of Medicine, Department of Pediatric Endocrinology, Ankara, Turkey

**Keywords:** GHRHR mutation, final height, transition, GH deficiency

## Abstract

Patients with growth hormone releasing hormone receptor (GHRHR) mutations exhibit pronounced dwarfism and are phenotypically and biochemically indistinguishable from other forms of isolated growth hormone deficiency (IGHD). We presented here two siblings with clinical findings of IGHD due to a nonsense mutation in the GHRHR gene who reached their target height in spite of late GH treatment. Two female siblings were admitted to our clinic with severe short stature at the age of 13.8 (patient 1) and 14.8 years (patient 2). On admission, height in patient 1 was 107 cm (−8.6 SD) and 117 cm (−6.7 SD) in patient 2. Bone age was delayed in both patients (6 years and 9 years). Clinical and biochemical analyses revealed a diagnosis of complete IGHD (peak GH levels on stimulation test was 0.06 ng/mL in patient 1 and 0.16 ng/mL in patient 2). Patients were given recombinant human GH treatment. Genetic analysis of the GH and GHRHR genes revealed that both patientscarried the GHRHR gene mutation p.Glu72X (c.214 G>T) in exon 3 in homozygous (or hemizygous) state. After seven years of GH treatment, the patients reached a final height appropriate for their target height. Final height was 151 cm (−1.5 SD) in patient 1 and 153 cm (−1.2 SD) in patient 2. In conclusion, genetic analysis is indicated in IGHD patients with severe growth failure and a positive family history. In spite of the very late diagnosis in these two patients who presented with severe growth deficit due to homozygous loss−of−function mutations in GHRHR, their final heights reached the target height.

**Conflict of interest:**None declared.

## INTRODUCTION

Growth hormone deficiency (GHD) is usually sporadic and may be a result of environmental cerebral insults or developmental anomalies. However, 3−30% of growth hormone (GH) deficient cases have an affected first−degree relative, suggesting a genetic etiology (1). Familial isolated growth hormone deficiency (IGHD) can result from genetic defects in genes encoding the GH, the GH secretagogue receptor, or the GH−releasing hormone receptor (GHRHR). It has been estimated that the mutations in the human GHRHR gene cause approximately 10% of autosomal recessive familial IGHD cases (2).

Patients with GHRHR mutations have marked dwarfism transmitted in a recessive fashion, and are phenotypically and biochemically indistinguishable from other forms of IGHD.

Regardless of etiology, early GH therapy in GHD children aims to prevent neonatal hypoglycemia and contribute to the attainment of better adult height. Age at initiation of GH treatment is one of the variables that influence final height (3).

We present here two siblings with clinical findings of IGHD due to a nonsense mutation in the GHRHR gene who responded well to GH therapy despite late admission.

## PATIENTS

**Figure 1:**
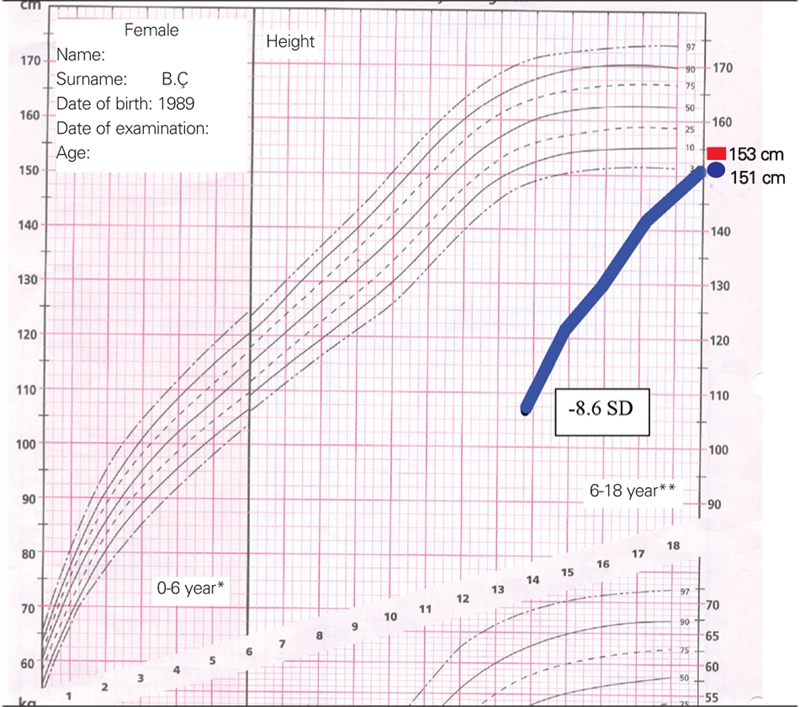
Two sister with GH−releasing hormone receptor (GHRHR) gene mutation: before treatment

Two female siblings were admitted to our clinic with severe short stature at the age of 13.8 (patient 1) and 14.8 years (patient 2) (Figure 1). On admission, patient 1 measured 107 cm (−8.6 SD) in height, and patient 2 117 cm (−6.74 SD). Bone age was delayed in both patients (corresponding to ages 6 years and 9 years at 13.8 and 14.8 years, respectively). Birth weight was 3000 g in patient 1 and 3200 g in patient 2. No history of neonatal hypoglycemia was reported. Their target height was 153 cm (−1.18 SD). Their parentally adjusted height deficits were −7.42 SD and −5.56 SD respectively.

Clinical examination revealed minimal midfacial hypoplasia with frontal bossing, depressed nasal bridge, abdominal obesity, high−pitched voice, and depressive behavior in addition to severe short stature (Figure 2). Both children were prepubertal and their level of intelligence appeared to be normal. Biochemichal analysis revealed very low levels of insulin−like growth factor−1 (IGF−1) and IGF−binding protein−3 (IGFBP−3), dyslipidemia, low basal GH, normal thyroid function tests, and normal levels of cortisol, adrenocorticotropic hormone (ACTH) and prolactin (Table 1). Insulin−induced hypoglycemia and L−Dopa stimulation tests were applied to the patients as GH stimulation tests. GH levels in response to stimulation tests were very low and set the diagnosis of severe complete IGHD in both patients.

Magnetic resonance imaging (MRI) showed anterior pituitary hypoplasia in both sisters. Treatment was initiated with recombinant human GH (rhGH) in a dose of 0.2 mg/kg/week administered as a daily subcutaneous injection. Chronological age was 13.9 years in patient 1 and 14.8 years−in patient 2.

During follow−up, onset of puberty was noted at 15.6 years in patient 1 and at 15.3 years in patient 2. Bone ages were 8 years 10 months in both patients at pubertal onset. Patient 2 received gonadotropin−releasing hormone analog (GnRHa) therapy in order to delay puberty and extend the beneficial effect of GH treatment on height gain. However, after one year of GnRHa treatment, no pubertal or bone age arrest was observed (bone age increased from 8 years 10 months to 12 years in one year), and GnRHa therapy was stopped. Menarche occurred at age 18.16 years in patient 1 and at 19.5 years in patient 2. Maternal menarcheal age was not precise. After seven years of GH treatment, patients reached their target height. Final height was 151 cm (−1.52 SD) in patient 1 and 153 cm (−1.18 SD) in patient 2 (Figure 2).

Genetic investigation included analysis of the GH and GHRHR genes, which revealed that both patients carry a GHRHR gene mutation p.Glu72X (c.214 G>T) in exon 3 in homozygous (or hemizygous) state.

**Figure 2:**
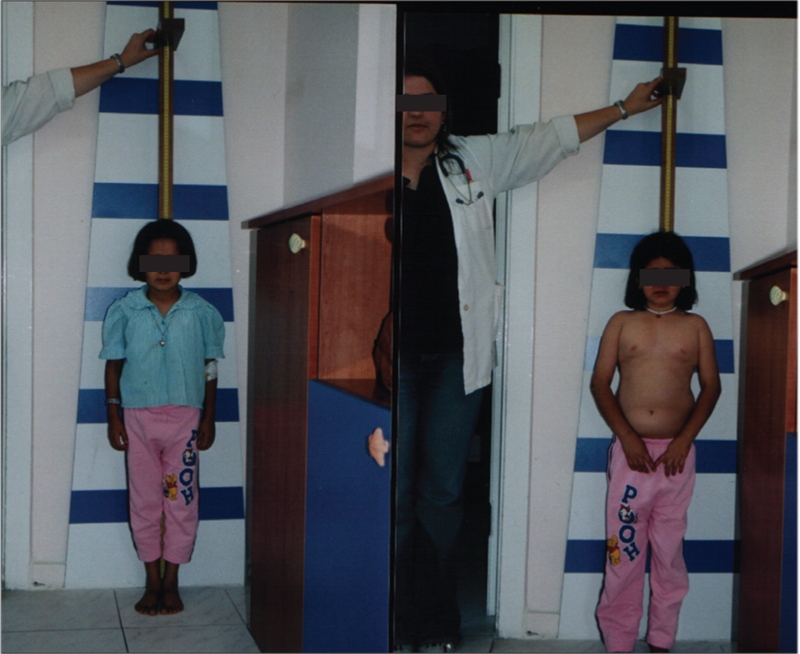
Two sister with GH−releasing hormone receptor (GHRHR) gene mutation: before treatment

**Figure 2:**
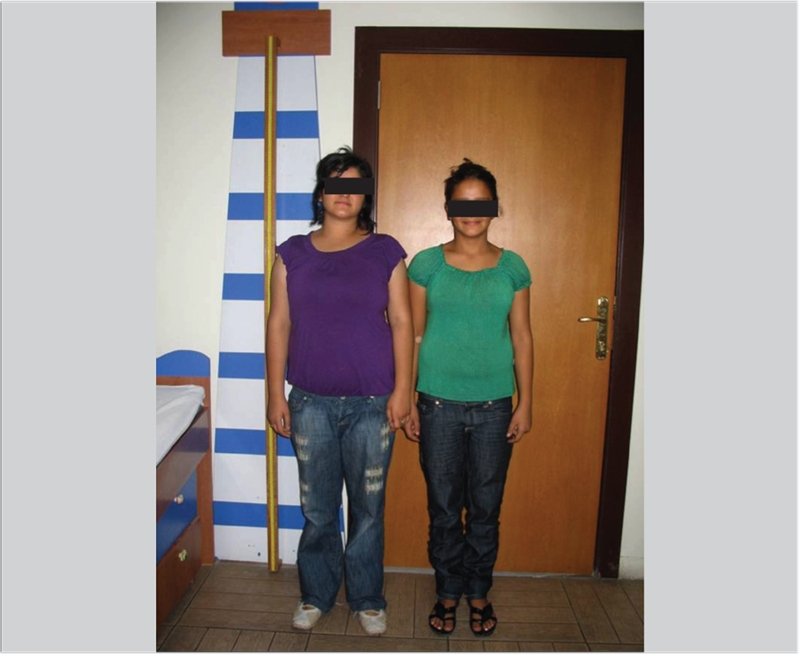
Patients with GH−releasing hormone receptor (GHRHR) mutation: after treatment

**Table 1:**
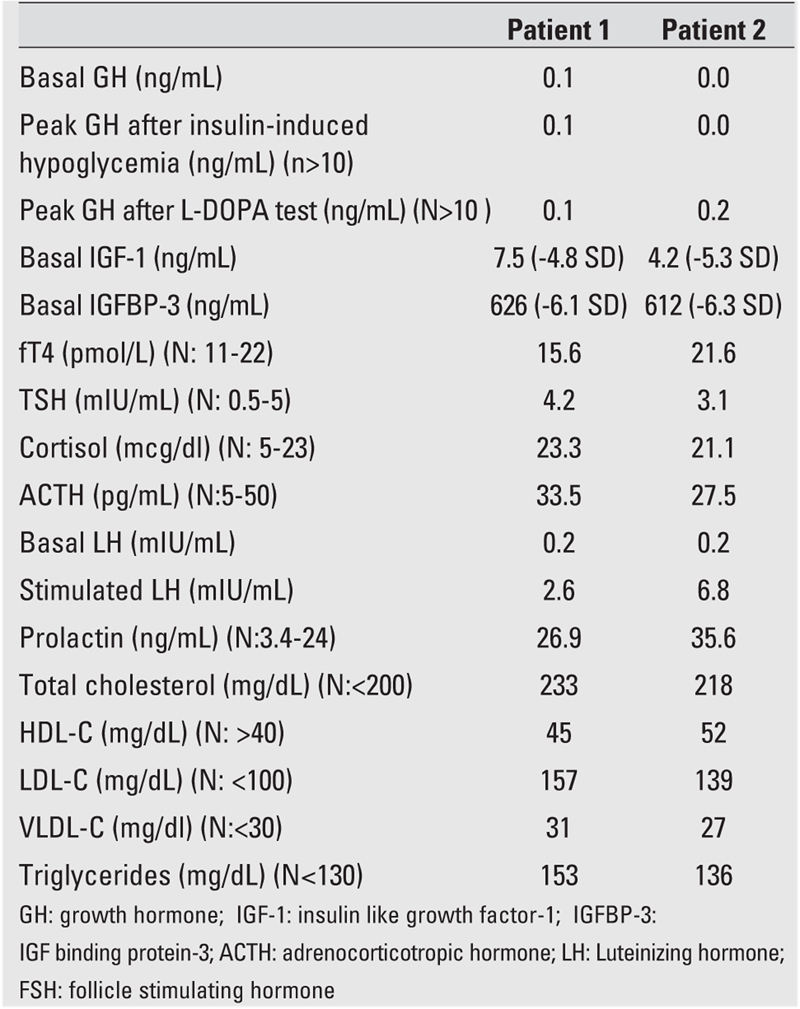
Biochemical analysis of patients on admission

## DISCUSSION

Familial IGHD is associated with at least four distinct forms (4). Two forms show autosomal recessive inheritance (IGHD type 1A and 1B), one form has autosomal dominant inheritance (IGHD type 2), and one is X−linked (IGHD type 3). Type 1B is the most frequently encountered form and could result from genetic defects in either GH gene or GHRHR gene (5). The GHRHR gene encodes a 423−aminoacid receptor protein and is essential for GHRH−stimulated secretion of GH (6).

GHRHR gene defects are recognized as the cause of approximately 10% of autosomal recessive IGHD cases (2). GHRHR mutations reported to date include six splice site mutations, two microdeletions, two nonsense mutations, seven missense mutations, and one in the promoter gene (7, 8, 9, 10). Our patients carried the GHRHR gene nonsense mutation p.Glu72X (c.214 G>T) in exon 3. Most cases with this same mutation originated from Asia, especially from India or Sri Lanka (11, 12).

Characteristics of patients with GHRHR mutations are very similar to those with GH gene defects. Patients carrying GHRHR mutations usually have high−pitched voices, increased abdominal fat, very short but normally proportioned stature, normal intelligence, minimal facial hypoplasia, very low levels of GH and IGF−1, and anterior pituitary hypoplasia on MRI (13, 14). Fertility is usually not affected, but puberty is reported to be delayed, especially in male patients (15). The female patients described herein showed similar characteristics of severe GHD.

Both patients had anterior pituitary hypoplasia on MRI. Mutations in GHRHR are usually associated with anterior pituitary hypoplasia but normally placed posterior pituitary on MRI. Given the important role of GHRH in regulating the proliferation and function of somatotroph cells, abnormalities in the GHRHR has been expected to cause anterior pituitary hypoplasia (13).

The aim of GH treatment is to avoid neonatal hypoglycemia and to attain better adult height. Our patients had no history of neonatal hypoglycemia. Late admission of patients with severe growth retardation is one important factor that compromises height increment. Early recognition of GHD is essential for an optimal height outcome (16). At admission, our patients were very short and their ages were not very young. It is known that final height is correlated with height for chronological age at diagnosis (3) the higher the chronological age, the lower the final height reached by the patients. One important factor in evaluating response to GH therapy is extent of attainment of the patient’s genetic targeted height. Our patients achieved their target height despite the very late age at diagnosis. In addition to age at diagnosis, compliance to GH therapy, late onset of puberty and slow pubertal maturation may be important factors in attainment of a satisfactory final height. Patient 1 entered puberty at age of 15.6 years and patient 2 − at 15.3 years. The duration of puberty was also normal, being 2.6 years in patient 1 and 4.3 years in patient 2. however use of GnRHa in patient 2 might have modified the duration of puberty. In addition, the severe degree of GHD in our patients may have affected the height gain. It is well known that growth rates correlate inversely with peak GH levels. The more severe the GH deficit is, the better the growth response is to GH (17).

In conclusion, genetic defects in related genes should be suspected in IGHD patients with severe growth failure and a positive family history, and molecular studies are indicated in such patients. In spite of the very late admission of these patients with severe growth deficit due to GHRHR gene mutations, their final heights reached the parentally adjusted height, probably because of delayed puberty. This observation indicates that prolonged GH replacement in patients with severe GHD will be beneficial, even when the diagnosis is made at older ages.
